# Molecular identification of *Coccidioides *spp. in soil samples from Brazil

**DOI:** 10.1186/1471-2180-11-108

**Published:** 2011-05-16

**Authors:** Regina CL de Macêdo, Alexandre S Rosado, Fabio F da Mota, Maria AS Cavalcante, Kelsen D Eulálio, Antônio D Filho, Liline MS Martins, Márcia S Lazéra, Bodo Wanke

**Affiliations:** 1 Laboratório de Micologia do Instituto de Pesquisa Clínica Evandro Chagas, Fundação Oswaldo Cruz, Rio de Janeiro, Brasil; 2 Laboratório de Ecologia Microbiana Molecular do Instituto de Microbiologia Paulo de Góes, Universidade Federal do Rio de Janeiro, Brasil; 3 Laboratório de Biologia Computacional e Sistemas, Instituto Oswaldo Cruz, Fundação Oswaldo Cruz, Brasil; 4 Universidade Estadual do Piauí, Teresina, Piauí, Brasil; 5 Universidade Federal do Piauí, Teresina, Piauí, Brasil

## Abstract

**Background:**

Since 1991 several outbreaks of acute coccidioidomycosis (CM) were diagnosed in the semi-arid Northeast of Brazil, mainly related to disturbance of armadillo burrows caused by hunters while digging them for the capture of these animals. This activity causes dust contaminated with arthroconidia of *Coccidioides posadasii*, which, once inhaled, cause the mycosis. We report on the identification of *C. posadasii *in soil samples related to outbreaks of CM.

**Results:**

Twenty four soil samples had their DNA extracted and subsequently submitted to a semi-nested PCR technique using specific primers. While only 6 (25%) soil samples were positive for *C. posadasii *by mice inoculation, all (100%) were positive by the molecular tool.

**Conclusion:**

This methodology represents a simple, sensitive and specific molecular technique to determine the environmental distribution of *Coccidioides *spp. in endemic areas, but cannot distinguish the species. Moreover, it may be useful to identify culture isolates. Key-words: 1. Coccidioidomycosis. 2. *Coccidioides *spp. 3. *C. posadasii*. 4. Semi-arid. 5. Semi-nested PCR

## Background

Coccidioidomycosis is a systemic mycosis acquired by inhalation of infective arthroconidia from *Coccidioides immitis *or *C. posadasii *[1], which are pathogenic species of dimorphic fungi that live saprobiotically in soil from arid regions of the western hemisphere [2]. The largest known endemic area covers the southwestern United States and all of semi-arid northern Mexico [3,4]. Coccidioidomycosis also occurs in several semiarid areas of Central and South America [5,6]. The most recent endemic area was discovered in Brazil, where the first two autochthonous cases acquired the infection in semi-arid regions of the states of Bahia and Piauí in 1978 and 1979. Since then, several cases have been diagnosed in these states and also in the states of Ceará and Maranhão [7,8].

*Coccidioides immitis *and *C. posadasii *are the only species included in the genus *Coccidioides*. These two species are morphologically identical but genetically and epidemiologically distinct [1,9]: *C. immitis *is geographically limited to California's San Joaquin valley, whereas *C. posadasii *is found in the remaining semi-arid areas in the southwest of the United States, Mexico, Central and South America.

Stewart & Meyer in 1932 reported the first isolation of *C. immitis *from soil, proving that this substrate is the primary source for coccidioidomycosis. They studied soil samples collected from a disturbed site in the San Joaquin river valley (California, USA) that was the possible source of an acute coccidioidomycosis outbreak [10]. Another important contribution to environmental studies on *Coccidioides *spp. was reported by Emmons in 1942, which was able to isolate the fungus from soil samples and from wild rodents in a known endemic area [11]. The fungus has been isolated by animal inoculation of a soil suspension in sterile saline, a method still considered to be gold standard for detecting fungus in environmental samples. As this method detects the parasitic spherule form in animal tissues, it permits the precise identification of *Coccidioides *spp. Unfortunately it is also an expensive methodology with relatively low sensitivity, and the results take a long time to obtain, usually up to 45 days [7,12,13].

The method of simply culturing soil samples on cycloheximide containing media slants is also very laborious, expensive, time consuming and of biological risk for the laboratory personnel. Comparing this method with that of animal inoculation, it is not able to demonstrate the parasitic form, necessary to ascertain the isolation of *Coccidioides *spp. [13].

In Brazil, the isolation of *Coccidioides *spp. from soil by animal inoculation has been used in some environmental investigations of small outbreaks of acute pulmonary coccidioidomycosis in armadillo hunters who are used to dig armadillo's burrows. Soil samples were collected inside and around armadillo's excavated burrows, ten to twenty samples, covering a small area of 4 to 6 m^2^. This method demonstrated the fungus in around 15% of the soil samples and it is important to emphasize that negative samples were often collected a few centimeters away from the positive ones. Thus, it is possible that viable elements of *C. posadasii*, with low metabolic activity and/or with low virulence, may be present in a soil sample but remain undetected by culture [7,14].

In the county of Oeiras, Piauí state, *C. posadasii *was isolated from three (12.5%) out of 24 soil samples collected in and around an excavated armadillo (*Dasypus novemcinctus*) burrow [7]. The same group of investigators obtained more environmental isolates of *C. posadasii *from soil samples related to excavation of armadillo (*D. novemcinctus*) and paca (*Cuniculus paca*) burrows in the county of Miguel Leão, Piauí [15-17].

Using multiplex PCR with two molecular markers, Greene et al (2000) demonstrated the presence of *C. immitis *in only four (0.55%) out of 720 soil samples collected in endemic areas of coccidioidomycosis in California (USA) [12].

The molecular identification of *Coccidioides *spp. in environmental samples depends on several factors, especially the sampling site, storage conditions, processing techniques, DNA extraction methods, and adequate choice of the genetic target.

There is a growing need in the knowledge of the global geographical distribution of *Coccidioides *spp., their focal distribution in endemic areas and their genetic diversity in the environment. In fact the development of efficient molecular tool for the environmental identification of *Coccidioides *spp. is a continuous challenge in order to comprehend the ecology and biogeography of this important pathogen.

The present study aimed to detect *Coccidioides *spp. in soil samples, related to small outbreaks of CM, by culture and molecular methods.

## Methods

The study was approved by the Institutional Ethics Committee of the Center for Biological Evaluation and Care of Research Animals at Fiocruz, no. P.0173-03 (COBEA at FIOCRUZ).

### Environmental soil sampling

Twenty-four soil samples were collected from two different sites suspected to be contaminated by *C. posadasii *in the counties of Caridade do Piauí (7°43'59''S, 40°59'23''W) and Elesbão Veloso (6°12'07''S, 42°08'25''W), situated 447 km and 156 km, respectively, from Teresina, the capital of the state of Piauí, in the northeast region of Brazil, which includes a vast semi-arid area. Soil samples were collected, in both sites, in burrows that were dug by the hunters who presented acute respiratory CM 9 to 14 days after the risk activity. Ten soil samples were collected in Elesbão Veloso (EV1-EV10) and 14 were collected in Caridade do Piauí (CP01, CP07, CP09 and CP12-CP22). The samples were placed into 100 mL sterile bags to be processed in Rio de Janeiro, at the Mycology Laboratory of IPEC/FIOCRUZ, according to both protocols: 1) animal inoculation in mice and 2) molecular detection. All soil samples were kept at room temperature (ranging from 20 to 28°C) till the arrival at FIOCRUZ in Rio de Janeiro.

As negative soil controls, eight environmental samples were collected in the savanna of central Brazil: four in Goiânia (LL 2611, 19 261101, V 2611 e C 261101) and four in Brasília (DF21, DF22, DF23 e DF24).

### Animal inoculation

The soil samples were processed and analyzed according to the classical technique described by Stewart & Meyer (1932), modified as follows: samples were weighed, and 1 g was mixed in 50 mL of 0.9% sterile saline with chloramphenicol (500 mg/L). Each suspension was vortexed and allowed to settle for 30 minutes at room temperature (25°C). The supernatant was aspirated, and 1 mL was inoculated intraperitoneally into four albino Swiss mice weighing 18-20 g. One control animal was used for each soil sample [10]. Animal necropsy was performed after four weeks of incubation or immediately after an animal death before the end of the incubation period. The animals were sacrificed in a CO_2 _chamber according to recommendations of COBEA. Liver and spleen samples were processed for a) direct mycological microscopy in wet mount preparations with 10% KOH; b) culture by inoculation onto Sabouraud 2% glucose agar medium DIFCO^® ^with and without cycloheximide; and c) preservation in 10% formalin for histopathological study. Control animals were not inoculated, but were maintained in a separate cage and subsequently submitted to the same protocol as the inoculated animals. This method is considered the gold standard for the isolation and identification of culture isolates suspected of being *C. immitis *or *C. posadasii*.

### DNA extraction from soil

The DNA was obtained using the Fast DNA^® ^SPIN^® ^Kit for soil (Q-BIOgene, Carlsbad, CA, USA) following the manufacturer's instructions. Soil DNA was analyzed by electrophoresis in 0.8% (w/v) agarose gels in Tris-Borate-EDTA buffer as well as in a spectrophotometer at 260 nm absorbance (Beckman DU-600) to check its amount, purity and molecular size. Final DNA obtained from soil samples had large molecular length (> 10 kb) and the humic acids contamination was not observed in electrophoresis gel. Therefore, DNA samples could be used as template to amplify 28S rDNA by PCR. DNA extracts were amplified by Polymerase chain reaction (PCR) using 1 μl of the extract (5 to 10 ng of DNA g soil^-1^) per 50 μl of reaction.

### Characterization of soil-extracted DNA

Soil-extracted DNA was amplified using the universal primers U1 and U2, which amplify a 260-bp product of a subunit of 28S fungal rDNA, to demonstrate the absence of PCR inhibitors and the presence of fungi in the sample, as described previously [18]. A negative control without DNA was included in all amplifications.

### DNA extraction from clinical and environmental isolates of *Coccidioides *spp

DNA of 21 clinical and environmental isolates of *Coccidioides *spp. was included in this study. From the Fungal Culture Collection at IOC/FIOCRUZ, six were identified as *C. immitis *(USA) and two as *C. posadasii *(Argentina); thirteen (nine clinical and four environmental) isolates identified as *C. posadasii *from Piauí/Brazil were preserved at the Laboratory of Mycology at IPEC/FIOCRUZ [19].

### DNA of other species of fungi and bacteria

DNA of several species of fungi (41) and bacteria (3) were included in the study: *Sporothrix schenckii *(5); *Paracoccidioides brasiliensis *(5); *Histoplasma capsulatum *(2); *Aspergillus niger *(3); *Aspergillus fumigatus *(3); *Aspergillus nidulans *(3); *Blastomyces dermatitidis *(1); *Microsporum canis *(1); *Trichophyton rubrum *(1); *Trichophyton mentagrophytes *(1); *Cryptococcus neoformans *(6); *C. gattii *(10); *Rhodococcus equi *(1); *Mycobacterium avium *(1); and *Paenibacillus sp*. strain 9500615. The isolates were preserved at the Laboratory of Mycology at IPEC/FIOCRUZ or obtained from soil samples preserved at the *Laboratório de Ecologia Microbiana Molecular *of IMPPG/UFRJ.

### Design of specific primers for *Coccidioides*

Initially, a BLASTn [20] search of GenBank revealed that the specific probe (5'-TCTGGCGGTTGGTT-3') for *C. immitis *proposed by Sandhu et al. (1995) presents 100% similarity with three *C. immitis *28S rDNA sequences deposited in the database [18]. However, this probe also presents 100% similarity with more than two hundred sequences of several other soil fungi and bacteria, leading the development of a new probe specific for *Coccidioides*. To obtain this new probe, all the 28S rDNA sequences of *Coccidioides *spp. and all other fungi deposited at GenBank until June 22, 2010, were aligned using the CLUSTAL X software [21]. Probes were designed based on conserved sequences of *Coccidioides *spp., and BLASTn software was used to identify specific probes for *Coccidioides *[20]. A probe designated RFA12 (5'-TCCCCCATGCTCCGGGCC-3') presented 100% sensitivity and specificity for all 22 sequences of *Coccidioides *(8 of *C. immitis *and 14 of *C. posadasii*) deposited at GenBank until June 2008 and was used together with an previously described probe P2 (5'-CTCTGGCTTCACCCTATTC-3') [18] to amplify a fragment of *Coccidioides *28S rDNA of around 375 bp. It was also evaluated the efficiency of a semi-nested PCR system, by using the pair of primers RFA12 and RFA13 (5'-TAATCATTCGCTTTACCTCA-3') which amplify a fragment around 520 bp, in a step before the using of RFA12 and P2 primers.

### Standardization of PCR from soil samples

To standardize a sensitive and specific molecular tool for detecting *Coccidioides *spp. in soil, the following steps were performed:

### PCR for cultured microorganisms

The PCR reaction mixture consisted of 1 μl of genomic DNA suspended in a mixture 5 μl 10 × PCR buffer (10 mM Tris (pH 9.0), 500 mM KCl), 2.5 μl of 10 mM dNTPs, 5 μl 25 mM MgCl_2_, 1 μl of each primer (RFA12/P2; 10 pmol/μl), 1.25 μl of 5 U AmpliTaq DNA polymerase, and 33.25 μl of MilliQ water. PCR amplification was performed with the primers (RFA12/P2) in a DNA thermal cycler. The temperature profile included an initial denaturation step at 94°C for 5 min; 30 cycles of 94°C for 30 s, 55°C for 1 min 30 s, and 72°C for 1 min; followed by a single terminal extension at 72°C for 3 min. As negative control, water instead of template was performed at all PCR reactions.

### Semi-nested PCR for cultured microorganisms

The reaction mixture of the the primary round PCR (RFA12/RFA13) consisted of 1 μl of DNA extract in a total volume of 50 μl with 5 μl 10 × PCR buffer (10 Mm Tris (pH 9.0), 500 mM KCl), 2.5 μl 10 mM dNTPs, 5 μl 25 mM MgCl_2_, 1 μl of each primer (10 pmol/μl), 1.25 μl of 5 U AmpliTaq DNA polymerase, and 33.25 μl of MilliQ water. The reaction cycles included an initial denaturation step at 94°C for 5 min; 20 cycles of 94°C for 30 s, 55°C for 1 min 30 s, and 72°C for 1 min; followed by a single terminal extension at 72°C for 3 min. Reaction mixtures of 2° PCR round (RFA12/P2) was identical, except by primers and 1 μl of the first reaction was added as template to the second reaction. Reaction mixtures with second primer set (RFA12/P2) were thermally cycled once at 94°C for 5 min, 30 times at 94°C for 30 s, 55°C for 1 min 30 s, and 72°C for 1 min, followed by a single terminal extension at 72°C for 3 min.

### PCR of soil

The reaction mixture of the primary PCR consisted of 1 μl of DNA extract in a total volume of 50 μl with 5 μl 10 × PCR buffer (10 mM Tris (pH 9.0), 500 mM KCl), 1 μl 10 mM dNTPs, 2.5 μl 50 mM MgCl_2_, 1 μl of each primer (RFA12/P2; 10 pmol/μl), 0.5 μl 10 mg/μl BSA, 0.5 μl 100% formamide, 0.5 μl of 5 U AmpliTaq DNA polymerase and 37 μl MilliQ water. The reaction cycles included an initial denaturation step at 94°C for 5 min, 35 cycles at 94°C for 45 s, 55°C for 1 min 30 s, and 72°C for 2 min, followed by a single terminal extension at 72°C for 3 min.

### Semi-nested PCR from soil

The reaction mixture of the primary round PCR (RFA12/RFA13) consisted of 1 μl of DNA extract in a total volume of 50 μl with 5 μl 10 × PCR buffer (10 mM Tris (pH 9.0), 500 mM KCl), 1 μl 10 mM dNTPs, 2.5 μl 50 mM MgCl_2_, 1 μl of each primer (10 pmol/μl), 0.5 μl 10 mg/μl BSA, 0.5 μl 100% formamide, 0.5 μl of 5 U AmpliTaq DNA polymerase and 37 μl MilliQ. The reaction cycles included an initial denaturation step at 94°C for 5 min, 25 cycles of 94°C for 45 s, 55°C for 1 min 30 s, and 72°C for 2 min, and a single terminal extension at 72°C for 3 min. Reaction mixtures of 2° PCR round was identical, except by primers and that 1 μl of the first reaction was added as template to the second reaction. Reaction mixtures with second primer set (RFA12/P2) were thermally cycled once at 94°C for 5 min, 35 times at 94°C for 45 s, 55°C for 1 min 30 s, and 72°C for 2 min, and a single terminal extension at 72°C for 3 min. A negative control without DNA was included in all amplifications.

### Evaluation of sensitivity of the semi-nested PCR

The sensitivity of the semi-nested PCR method was determined with primers specific for *C. immitis *(RFA12/RFA13 and RFA12/P2) using DNA of a *C. posadasii *isolate, either pure (without dilution) or diluted by 10^-2^, 10^-3 ^and 10^-4 ^in water free of DNAse and RNAse. Next, 0.5 μl of negative soil DNA (soil from an area without coccidioidomycosis) was added to 0.5 μl of each pure and diluted DNA sample in triplicate. All products obtained by direct PCR and semi-nested PCR were subjected to electrophoresis in a 1.2% agarose gel with 1 × TBE buffer (89 mM Tris-borate, 2.5 mM EDTA [pH 8.0]) for 2 h, and a 1 Kb DNA Ladder (Promega) served as molecular marker. The gel was then stained for 15 min with 0.5 μg ml^-1 ^ethidium bromide and observed under short-wavelength ultraviolet light. The image was captured by an IMAGO system.

## Results

### Animal inoculation

*C. posadasii *was isolated by intraperitoneal inoculation into mice, from 6 (25%) out of the 24 soil samples studied: 3 out of 10 (30%) from Elesbão Veloso and 3 out of 14 (21.4%) from Caridade do Piauí.

### Molecular methods

The DNA obtained from each soil sample was of excellent quality, with its molecular weight concentrated above 1.5 kb

### PCR and semi-nested PCR applied to DNA of cultured of Coccidioides spp. and controls

**Figure 1:**
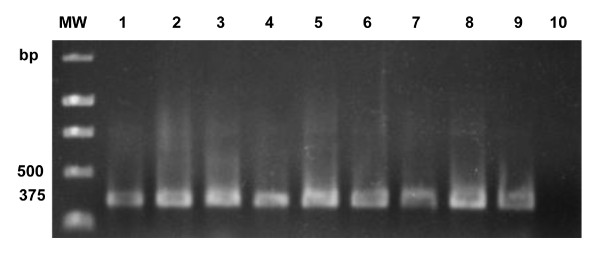
**1.2% agarose gel showing results of semi-nested PCR with primers RFA12/RFA13 and RFA12/P2 specifics for *Coccidioides *spp., lines 1-4 DNA isolated of *C. immitis *(US), lines 5-9 DNA isolated of *C. posadasii *(Piauí/Brazil), and line 10 negative control (DNA *C. neoformans*)**. MW = 1 Kb DNA Ladder (Promega).

**Figure 2:**
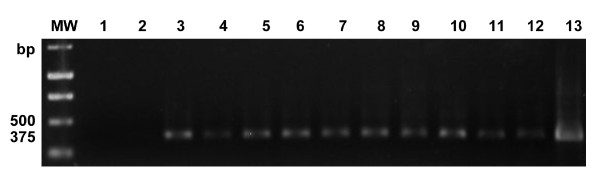
**1.2% agarose gel showing results of semi-nested PCR with primers RFA12/RFA13 and RFA12/P2 specifics for *Coccidioides *spp. lines 1-2 DNAs *Rhodococcus equi *33701 e *Mycobacterium avium *13956, lines 3-4 DNA isolated of *C. immitis *(US), lines 5-6 DNA isolated of *C. posadasii *(Argentina) and lines 7-13 DNA isolated of *C. posadasii *(Piauí/Brazil) MW = 1 Kb DNA Ladder (Promega)**.

Direct PCR with primers specific for *Coccidioides *spp. (RFA12/P2) was able to identify 19 out of the 21 *Coccidioides *spp. isolates tested, which presented the specific 375-bp band. However, semi-nested PCR using the same primers, RFA12/RFA13 and RFA12/P2, was able to identify all the 21 isolates tested (Figures 1 and 2). The same direct and semi-nested PCR methodologies presented negative results when applied to DNA of all species of other different pathogenic fungi and bacteria. These results demonstrate the high specificity of the primers developed in this study and highlight the increased sensitivity, expected in semi-nested PCR reactions from environmental samples.

### PCR and semi-nested PCR applied to soil DNA samples

**Figure 3:**
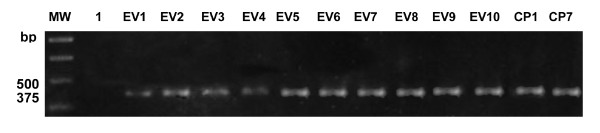
**1.2% agarose gel showing results of semi-nested PCR with primers RFA12/RFA13 and RFA12/P2 specific for *Coccidioides *spp., lines 2-11 soil samples from Elesbão Veloso (EV), lines 12 and 13 Caridade do Piauí (CP)**. Line 1 = white and MW = 1 Kb DNA Ladder (Promega).

The DNA obtained from the soil samples was submitted to direct PCR and semi-nested PCR using the same primer system. Only 8 out of 24 (33.3%) soil samples presented the specific 375-bp band by direct PCR: 2/10 from Elesbão Veloso and 6/14 from Caridade do Piauí (Data not shown). However, using semi-nested PCR with the primers RFA12/RFA13 and RFA12/P2, all the soil samples presented the specific 375-bp band indicative of *Coccidioides *spp. (Figure 3). By the same molecular method, the DNA obtained from the soil of central Brazil presented 100% negative results. The results comparing both classical and molecular methods to detect *Coccidioides *spp. in soil samples are summarized in Table 1.

**Table 1:** Detection of *C. posadasii *in soil samples by classical and molecular methods in Piauí, Brazil.

Soil sample	Animal inoculation	Direct PCR	Semi-nested PCR
EV1	**-**	**-**	**+**
EV2	**-**	+	**+**
EV3	**-**	**-**	**+**
EV4	**-**	+	**+**
EV5	**-**	**-**	**+**
EV6	+	**-**	**+**
EV7	**-**	**-**	**+**
EV8	+	**-**	**+**
EV9	**-**	**-**	**+**
EV 10	+	**-**	**+**
CP1	**-**	**-**	**+**
CP7	**-**	**-**	**+**
CP9	**-**	**-**	**+**
CP12	**-**	**-**	**+**
CP13	**-**	**-**	**+**
CP14	**-**	**-**	**+**
CP15	**-**	**-**	**+**
CP16	**-**	**+**	**+**
CP17	**+**	**+**	**+**
CP18	**+**	**+**	**+**
CP19	**+**	**+**	**+**
CP20	**-**	**+**	**+**
CP21	**-**	**+**	**+**
CP22	**-**	**-**	**+**

EV = Elesbão Veloso, CP = Caridade do Piauí,

(-) = Negative and (+) = Positive

### Evaluation of the sensitivity of the semi-nested PCR for detecting specific sequences of *Coccidioides *spp

**Figure 4:**
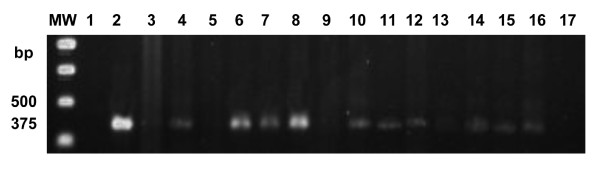
**1.2% agarose gel showing results of semi-nested PCR with primers RFA12/RFA13 and RFA12/P2 specific for *Coccidioides *spp., lines 1, 5, 9, 13 and 17 = white, lines 2-4 DNA *C*. *posadasii *(pure), lines 6-8 DNA *C. posadasii *(diluted at 10^-2^), lines 10-12 DNA *C. posadasii *(diluted a 10^-3^), lines 14-16 DNA *C. posadasii *(diluted 10^-4^)**. MW = 1 Kb DNA Ladder (Promega).

Semi-nested PCR with primers specific for *Coccidioides *spp. (RFA12/RFA13 and RFA12/P2) when applied to DNA of *C. posadasii *in serial dilutions was sufficiently sensitive to detect specific *C. immitis *28S rDNA, generating a product of 375-bp, as visualized in a 1.2% agarose gel (Figure 4).

## Discussion

Inoculation into mice has long been the classical method for isolating and identifying pathogenic fungi present in environmental samples such as soil. Many studies have been performed over several decades, mainly by intraperitoneal inoculation into albino, non-isogenic and non-immunocompromised mice, thereby producing knowledge on the geographic distribution, natural habitats and environmental microfoci of pathogenic fungi, especially *Histoplasma *and *Coccidioides *spp.

Due to its nature, the animal model works as a biological filter, selecting species or lineages thermo tolerant to 35 - 37°C with metabolic and genetic properties that permit their survival and multiplication in mammalian tissues. Usually, when suspected soil material is inoculated intraperitoneally, the saprobic microbiota composed of bacteria and fungi are blocked and eliminated by the immune system of the inoculated mice. In the presence of fungal agents of systemic mycoses, they may multiply and disseminate to regional lymph nodes and other organs like the lungs, liver, spleen, kidneys, skin and/or central nervous system. Spleen and liver were the organs that allowed the highest positivity for isolating *Coccidioides *spp. of the inoculated mice [10].

*Coccidioides *spp. isolates have been obtained from soil samples of known endemic areas. Usually, the positivity is very low when the samples are collected randomly, even in endemic areas; however, when sampling is directed to a specific suspected site related to cases of acute pulmonary coccidioidomycosis with a consistent epidemiological history of dust inhalation, the probability of obtaining positive samples increases significantly. In fact, such sites may harbor microfoci of *Coccidioides *spp. where they find suitable ecological conditions to multiply and reach high spore concentrations in restricted areas. These quantitative aspects have been demonstrated for *Cryptococcus neoformans *and *C. gattii *through plating onto selective Niger Seed agar (NSA) medium, which allows the concentration of viable fungal propagules to be estimated [22]. Nevertheless, this plating method is not used to detect the agents of coccidioidomycosis due to its high biological risk and because other fast-growing saprobic fungi may lead to misidentification. Thus, there are no adequate tools for estimating the concentration of *Coccidioides *spp. elements in various substrata, natural habitats or environmental sources related to outbreaks of coccidioidomycosis, where high concentrations of the fungus may exist.

The low frequency of *C. immitis *isolation from soil samples may be due to seasonal variations or a non-homogeneous distribution in the soil. A study conducted in the US investigated environmental samples collected over eight years in the same endemic area detected the presence of *C. immitis*, ranging from 0 - 43% [14]. Few environmental isolates of *C. immitis *and *C. posadasii *from endemic areas of Mexico and the United States are available for scientific purposes. Recent studies on the phylogeny and molecular epidemiology of *Coccidioides *spp. were based mainly on clinical isolates from different geographical regions [1,9]. Therefore, environmental isolates of *C. posadasii *from semi-arid northeastern Brazil are of interest for these studies.

Regarding the environmental samples collected in and around two excavated armadillo (*D. novemcinctus*) burrows in Elesbão Veloso and Caridade do Piauí, we obtained positivity rates of 30% and 21.4%, respectively, using the mouse inoculation method. These rates seem very satisfactory when compared to literature data Greene et al. 2000 [12]. The low number of soil samples collected in a specific contaminated habitat excavated during armadillo hunting may have contributed to these results. Moreover, it should be taken into consideration that only a small amount (1 g) from each soil sample was examined after suspending it in 50 mL of saline, from which only 0.5 mL was inoculated into each mouse. Thus, it is possible that viable propagules of *Coccidioides *spp. present in the sample were not inoculated, producing a false negative result. Beyond the quantitative aspect, the animal model is incapable of detecting lineages unable to grow at 37°C or present in numbers too low to invade and grow in mammalian tissues. On the other hand, propagules with low metabolic activity can remain in latency in soil. In fact, most aspects of the population structure of *Coccidioides *spp. in the environment remain unknown.

Curiously, during the investigation of the samples from Caridade do Piauí, the same method of animal inoculation permitted the simultaneous isolation of *C. posadasii *and *Cryptococcus neoformans *from one soil sample, while *C. neoformans *was isolated from another soil sample that was negative for *C. posadasii*. These findings demonstrate the complexity of the fungal microbiota in environmental habitats, such as in this case of *D. novemcinctus*. These habitats are not exclusive to armadillos, but they are shared with wild rodents, snakes, scorpions, birds and many insects. In the surroundings of this armadillo burrow, it was observed a resting site of *Zenaida auriculata*, a New World tropical dove endemic to South America, that appear periodically in this region. It is possible that *Coccidioides *spp., *Cryptococcus neoformans *and *C. gattii*, interacting together and/or with other living elements of the soil microbiota, as well as with several other hosts, may generate adaptations and select lineages of these pathogenic fungi. The demonstration of naturally acquired coccidioidomycosis in *D. novemcinctus *armadillos captured in Piauí reinforces the complexity of this subject [23]. Nevertheless, there have been no investigations of naturally acquired coccidioidomycosis in other species of armadillos, or in other animals such as rodents, foxes, goats, horses, donkeys, cattle and other mammals.

Molecular biological techniques have been used to identify pathogenic fungi. Sandhu et al. (1995) analyzed 116 cultures of several human pathogenic fungi using the universal primers U1 and U2 to amplify the conserved 28S rDNA region of fungi, which was then hybridized with probes specific for each fungal species [18]. Sixteen clinical isolates of *C. immitis *tested by this method demonstrated 100% positivity in identifying this species.

Another approach used for the identification of isolates of *C. immitis *is direct PCR using primers with nucleotide sequences based on the gene *csa*, which is a 19-kDa specific *C. immitis *antigen secreted in the growth phase of fungal cultures that generates a product of about 519 bp [24]. In another study, Bezerra et al. (2006) obtained 100% positivity analyzing the DNA of 19 cultures of *C. immitis*: twelve clinical isolates from the state of Piauí and seven isolates preserved for 50-75 years in the culture collection of the Department of Mycology from the Instituto Oswaldo Cruz at FIOCRUZ in Rio de Janeiro [19].

Regarding the development of molecular methods for the detection of *Coccidioides *spp. directly in soil samples, obtaining an adequate DNA preparation represented a large challenge. Using mechanical agitation followed by direct cellular enzymatic lysis, we obtained DNA samples with a molecular weight concentrated above 1.5 kb, which were suitable for the amplification reactions by PCR. It should be mentioned that only recently adequate equipment and a Fast DNA SPIN kit for soil (QBIOgene, Carlsbad, CA, USA) allowed the attainment of this suitable DNA from soil samples.

In the present study, the primers designed to detect *Coccidioides *spp. 28S rDNA in soil took into consideration the low number of copies of the target DNA present in soil. This permitted the detection of *Coccidioides *spp. 28S rDNA in six isolates from the USA and two from Argentina, as well as in thirteen Brazilian isolates. The molecular detection of any of the *Coccidioides *species in soil or in clinical specimens is of equal importance.

Optimization of direct PCR with specific primers to detect *C. immitis *was first performed with DNA extracted from 21 lineages of *Coccidioides *spp. (eight from the Fungal Culture Collection at IOC/FIOCRUZ, and nine clinical and four environmental isolates from the state of Piauí preserved at the Laboratory of Mycology of IPEC/FIOCRUZ). In this way, we detected a 28S rDNA fragment with a product of nearly 375 bp in 19 out of the 21 isolates tested. However, applying a semi-nested PCR system to these DNA samples with a new pair of primers specific for *Coccidioides spp.*, we detected bands of sizes compatible with the expected fragment in the DNA of all cultures tested. As a control, the DNA of 41 lineages of other human pathogenic fungi (*S. schenckii, P. brasiliensis, H. capsulatum, A. niger, A. fumigatus, A. nidulans*, *B. dermatitidis*, *M. canis*, *T. rubrum, T. mentagrophytes, C. neoformans *and *C. gattii*) were submitted to the same protocol, and all results were negative. The results were also negative when the protocol was applied to DNA from bacteria.

Our results indicate the high specificity of PCR with these primers and highlight the increased sensitivity, expected in nested PCR reactions using DNA obtained from soil samples. The next step was to optimize direct PCR with specific primers for detecting *Coccidioides *spp. in the DNA extracted from our 24 soil samples. The direct PCR method revealed the expected fragment only in 8 (33.3%) soil samples, but when the semi-nested system was used, all the soil samples were positive, thus confirming to be a very sensitive method for detecting *Coccidioides *spp. 28S rDNA. It is important to note that all of the positive soil samples were collected in and around armadillo burrows strongly suspected to be heavily contaminated because their disturbance caused acute cases of human and canine coccidioidomycosis. It is possible that these restricted sites harbor high concentrations of viable arthroconidia of *C. immitis*, which are easily detected by animal inoculation, as well as dormant or dead fungal elements with DNA partially preserved, which can only be detected by molecular tools. To evaluate these factors, it should be of interest to analyze soil samples collected in concentric circles from the center of the focus.

As controls for the PCR protocols applied to our soil samples from Piauí, we analyzed DNA extracted from soil samples collected in non-endemic areas of the cities of Goiânia (capital of the state of Goiás) and Brasília (Capital of Brazil), and none presented the 375-bp band, reinforcing our results. Thus, we believe it is important to note that the primer system RFA12 + P2 was able to identify both *C. immitis *and *C. posadasii*.

The molecular detection of *Coccidioides *spp. in suspected soil or in clinical specimens has obvious importance for epidemiological studies and laboratory diagnosis of coccidioidomycosis. Furthermore, molecular procedures such as PCR present substantial advantages, as they reduce the biological risk inherent in the classical techniques and reduce the time necessary to identify a suspected environmental focus or diagnose a clinical case to a few hours. On the other hand, this 28S rDNA marker is not able to distinguish *C. immitis *from *C. posadasii *in positive soil samples. However, other markers can be used to detect these specific species. Umeyama et al. (2006) describe species-specific primers for *C. immitis *based on the ITS1 and ITS4 region, and they were able to differentiate isolates of *C. immitis *and *C. posadasii *[25] .

The methodology described in the present study was found to be a sensitive and specific tool for detecting *Coccidioides *spp. in soil. We believe that the RFA12 + P2 primer system will be useful for epidemiological investigations of clinical cases as well as for environmental studies to identify hazardous sites in Brazil and elsewhere.

## Conclusions

This study introduced a simple, sensitive and specific molecular technique to determine the environmental distribution of *Coccidioides *spp. in endemic areas, but cannot distinguish the species.

## Abbreviations

COBEA at FIOCRUZ: Institutional Ethics Committee of the Center for Biological Evaluation and Care of Research Animals at Fiocruz; CM: Coccidioidomycosis; EV: Elesbão Veloso; CP: Caridade do Piauí; IPEC: Instituto de Pesquisa Clínica Evandro Chagas; DF: Distrito Federal; IOC: Instituto Oswaldo Cruz; FIOCRUZ: Fundação Oswaldo Cruz

## Authors' contributions

RCLM: Study design, primers design, fieldwork and data collection, laboratory tests, data analysis, manuscript writing; ASR: Study design, primers design, laboratory tests, data analysis, manuscript writing; FFM: Primers design, laboratory test, data analysis, manuscript writing; MASC: Fieldwork, data collection and analysis, manuscript writing; KDE: Fieldwork and data collection; ADF: Fieldwork and data collection; LMSM: Diagnostic laboratorial tests; MSL: Data interpretation and analysis, manuscript writing; BW: Coordination, study design, fieldwork and data collection, data analysis, manuscript writing. All authors read an approved the final draft.

## Authors' information

RCLM: regina.macedo@ipec.fiocruz.br

ASR: asrosado@globo.com

FFM: fabio@ioc.fiocruz.br

MASC: normacely@uol.com.br

KDE: kelsendeulalio@yahoo.com.br

ADF: mdedeus@uol.com.br

LMSM: liline-martins@uol.com.br

MSL: marcia.lazera@ipec.fiocruz.br

BW: bodo.wanke@ipec.fiocruz.br
